# Preparation and Characterization of Fluorinated Acrylate and Epoxy Co-Modified Waterborne Polyurethane

**DOI:** 10.3390/polym16182576

**Published:** 2024-09-12

**Authors:** Yufei Zhao, Shuai Yang, Jianjun Zhang, Shaoxiong Xu, Jinhui Han, Sude Ma

**Affiliations:** 1 College of Materials Science & Engineering, Xihua University, Chengdu 610039, China; zhaoyufei0930@163.com (Y.Z.); zjjxjtu@163.com (J.Z.); xsx281596@163.com (S.X.); hanjinhui0813@163.com (J.H.); 2 Laboratory of Advanced Energetic Materials and Devices, Xihua University, Chengdu 610039, China; 3 Dongfang Electric Machinery Co., Ltd., Deyang 618000, China; langhaiyang@126.com

**Keywords:** waterborne polyurethane, fluorinated block copolymer, hydrophobic film, fluorinated acrylate, epoxy co-modified

## Abstract

Conventional waterborne polyurethane (WPU) has poor water resistance and poor overall performance, which limits its application in outdoor coatings. A solution to this problem is urgently needed. The introduction of fluorine-containing groups can effectively improve the water resistance of WPU. In this study, a new fluorinated chain extender (HFBMA-HPA) synthesized by free radical copolymerization and epoxy resin (E-44) were used to co-modify WPU, and five waterborne fluorinated polyurethane (WFPU) emulsions with different fluorine contents were prepared by the self-emulsification method. The effects of HFBMA-HPA content on the emulsion particle properties, coating surface properties, mechanical properties, water resistance, thermal stability, and corrosion resistance were investigated. The results showed that the WFPU coating had excellent thermal stability, corrosion resistance, and mechanical properties. As the content of HFBMA-HPA increased from 0 wt% to 14 wt%, the water resistance of the WFPU coating gradually increased, the water contact angle (WCA) increased from 73° to 98°, the water absorption decreased from 7.847% to 3.062%, and the surface energy decreased from 32.8 mN/m to 22.6 mN/m. The coatings also showed impressive performances in the adhesion and flexibility tests in extreme conditions. This study provides a waterborne fluorinated polyurethane material with excellent comprehensive performance that has potential application value in the field of outdoor waterproof and anticorrosion coatings.

## 1. Introduction

A waterborne polyurethane (WPU) is a material that contains repeating urethane groups and is dispersed in water. Compared with traditional solvent-borne polyurethanes, WPU exhibits excellent flexibility, environmental friendliness, and superior physical and chemical properties, and is thus widely applied in various industries [1,2,3,4,5]. Nevertheless, the introduction of an extensive number of hydrophilic components during the synthesis of WPU leads to poor mechanical characteristics and water resistance of the coating, which restricts the application scenarios of the material [6,7,8,9].

Fluorine is an extremely electronegative element with a weak polarizability and high tendency to self-aggregate, and the fluorine atoms in organic fluorinated compounds are distributed in a helical pattern along the C-C single bonds [10,11]. On the other hand, the bond energy of the C-C bond is lower than that of the C-F bond, and the electron cloud of fluorine atoms has a stronger shielding effect on the C-C bond [12], whose properties give fluoropolymers low surface energy, excellent self-cleaning properties, and outstanding thermal stability [13,14,15,16]. Therefore, fluoropolymers were introduced into the WPU system to obtain fluorinated waterborne polyurethane (WFPU), which combined the advantages of WPU and fluoropolymers. The water resistance, weatherability, antifouling, and thermal stability of WPU were improved more efficiently [17].

Several methods of introducing fluoropolymers into the WPU molecular chain were introduced. Firstly, fluorinated diisocyanatos were introduced into the hard section of the WPU molecular chain [18,19]. However, they are rarely used due to their high cost, limited availability, and restricted industrial applications [20]. Secondly, fluorinated monohydric alcohols [21] were used at the end of the WPU molecular chain to cap the WPU. This introduced a limited amount of fluorine content, and the enhancement of WFPU properties was less significant [22]. Thirdly, fluorinated diols [23] or polyether diols [24] were introduced in the soft segment of the WPU molecular chain. The migration of fluorine-containing groups was limited by the large molecular chain, and fluorine atoms were not sufficiently enriched on the coating surface [19]. For example, Hwang et al. [24] developed a UV-curable fluorinated polycarbonate-based polyurethane dispersion using perfluoropolyether (PFPE) as the soft segment and the resulting material exhibited a low contact angle of only 93.3°. Chen et al. [25] utilized 100% renewable polytetramethylene ether glycol and PFPE to prepare a series of fluorinated waterborne polyurethanes (FWPUs) via self-emulsification. The WCA of the films increased from 80.8° to 100° as the PFPE content was increased from 0 wt% to 15 wt%. This was attributed to the fact that the immobilized PFPE in the main chain of FWPU limited the migration of fluorine atoms to the coating surface. Fourth, fluorinated chain extenders [6,16] usually introduce fluorinated groups into the WPU in the form of side chains, and the fluorinated groups are less affected by intermolecular interactions within the main chain. This can make it easier for fluorine atoms to migrate towards the coating surface, thus significantly improving the performance of WFPU coatings and broadening their application range. Therefore, fluorinated chain extenders have received increasing attention from researchers [26,27,28]. Li et al. [6] used diallyl bisphenol A (DBA) and 1H,1H,2H,2H-perfluorodecanethiol (PFDT) as the raw materials to synthesize a fluorinated diol with fluorine groups in its lateral chain (DFP) via a thiol–ene click reaction. Subsequently, it was embedded in PU as a chain extender to obtain highly hydrophobic (120°) and oleophobic (121°) coatings. However, relatively few studies have been conducted on the co-improvement of hydrophobicity and mechanical properties of WPU coatings.

Previous studies have shown that the incorporation of epoxy resins into WPU systems can effectively increase the degree of crosslinking of the WPU molecular chains and thus improve the mechanical properties of the coatings [29,30,31]. Epoxy resin has been extensively employed in the domains of electronics, aerospace, adhesives, and coatings owing to its superior physical properties, elevated bonding strength, corrosion preventive, electrical insulation, and well-processed processability [32,33,34]. Among all types of epoxy resins, the bisphenol A-based epoxy resin is designated as the general purpose epoxy resin owing to its readily accessible raw materials, lowest cost, largest production scale, and broadest applications [35,36,37]. Therefore, in order to prepare low-cost WFPU coatings, we considered the use of a bisphenol A-type epoxy resin (E-44) and a homemade chain extender with fluorine groups in the lateral chain to improve the mechanical properties and hydrophobicity of the coatings. However, the cost of fluorinated materials remains high. Although synthesis technology is improving, the synthesis process of WFPU is still relatively complex. WFPU is more environmentally friendly than solvent-based polyurethanes, but some environmentally harmful by-products may still be generated in the production process. As research on WFPU continues, its synthesis method, structure, and properties are being widely studied. It is expected that a WFPU with lower cost, better performance, and environmental friendliness can be developed in the future, and is expected to contribute to more fields, such as biomedicine and aerospace.

In this work, random fluorine-containing block copolymers (HFBMA-HPA) with fluorine groups in the lateral chain were synthesized through a simple free-radical copolymerization method with relatively low-priced 2,2,3,4,4,4-hexafluorobutyl methacrylate (HFBMA) and β-hydroxypropyl acrylate (HPA) as raw materials, which were introduced as chain extenders into the soft segments of polyurethanes. The fluorine-containing groups were introduced into the main chain of the polymer as lateral chains, which reduced the influence of the main chain on the enrichment of fluorine on the surface and enhanced the migration efficiency of the fluorine within the material. Concurrently, E-44 was employed as a crosslinking agent to modify the polyurethane to increase the degree of crosslinking of the molecular chain, thereby improving the mechanical properties of the polyurethane. The rationally designed and synthesized WFPU coatings with excellent mechanical characteristics and hydrophobicity were comprehensively evaluated for their water resistance, thermal stability, and corrosion resistance. This study presents a new strategy to produce waterborne polyurethanes with low cost, excellent hydrophobicity, water resistance, and mechanical properties.

## 2. Experimental

### 2.1. Materials

Isophorone diisocyanate (IPDI) was supplied by Shanghai Aladdin Industrial Corporation, China. HFBMA was provided by Harbin Xuejia Fluorosilicon Co., Ltd. in Harbin, China. Polyethylene glycol (PEG) with an average molecular weight of 200 g mol^−1^ and 4000 g mol^−1^ (both vacuum pre-dried at 80 °C for 2 h), azobisisobutyronitrile (AIBN), HPA, dibutyltin dilaurate (DBTDL), acetone, and methyl ethyl ketone were all provided by Chengdu Kelong Chemical Co., Ltd. in Chengdu, China. Epoxy resin (E-44) was supplied by Shandong Yousuo Chemical Technology Co., Ltd. in Linyi, China. CYML 385 and BECKOPOX^TM^ EH 613 w/80 WA curing agents (mixed well at 17:3 mass ratio before use) were supplied by Zhanxin Resin Co., Ltd. in Shanghai, China.

### 2.2. Preparation of HFBMA-HPA

**Figure 1:**
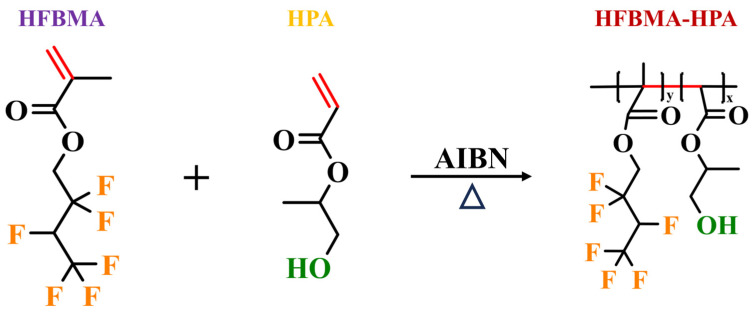
Synthesis process for HFBMA-HPA.

HFBMA-HPA was synthesized through free radical polymerization. The synthesis route of HFBMA-HPA is depicted in Figure 1. First, a solution of HFBMA and HPA (molar ratio 2:1) was made as solution A. Then, the initiator AIBN (3% of the mass of solution A) was dissolved in a certain amount of methyl ethyl ketone to form solution B. Solution A and B were then mixed and added to a four-necked flask. The reaction was carried out at 75 °C under N_2_ atmosphere with magnetic stirring for 14 h, during which the methyl ethyl ketone was recovered using a spherical condenser. After completion of the reaction, the four-necked flask was cooled to room temperature to obtain the HFBMA-HPA product. The unreacted monomers and methyl ethyl ketone were then removed by vacuum distillation.

### 2.3. Preparation of WFPU

**Figure 2:**
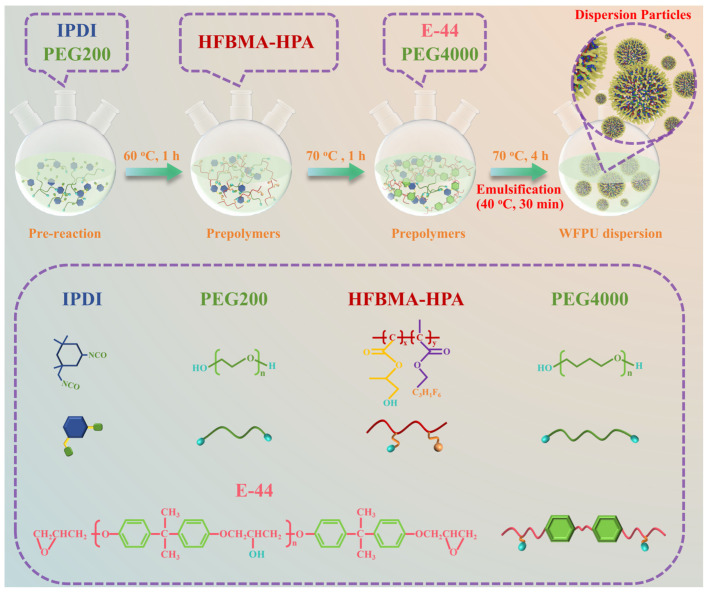
Synthesis process for WFPU.

The synthesis route of WFPU is shown in Figure 2 and WFPU synthesis and the characterization overview diagram are shown in Figure S1. A fluorine-containing emulsion was prepared in four steps using the acetone method. First, IPDI, PEG200, and DBTDL were mixed and added to a four-necked flask equipped with a spherical condenser and stirrer. The mixture was stirred at 60 °C under N_2_ atmosphere for 1 h. Then, the temperature was increased to 70 °C, and HFBMA-HPA was added and reacted for 1 h. PFG4000 and E-44 were then added, and the reaction was continued for 5 h. Finally, the temperature was decreased to 40 °C, and deionized water was slowly added dropwise under high-speed stirring (1000 rpm) for 30 min to emulsify the system. Acetone was added during the reaction to control the system viscosity. After the reaction, the acetone was removed by vacuum distillation to obtain the target product. Using this method, 5 WFPU-n emulsions were prepared, where n represents the mass percentage of HFBMA-HPA in the solid component of the emulsion (0, 3.5, 7, 10.5, 14 wt%). The monomer compositions of the WFPU-n syntheses are shown in Table 1.

### 2.4. Preparation of WFPU-n Film and Coating

In accordance with the national standard GB/T 1727-2021 [38], a suitable quantity of the prepared emulsion was taken, and 30 wt% of curing agent was added to its solid constituent. The two were then uniformly mixed using an ultrasonic machine, and evenly sprayed onto the surface-treated tinplate employing a high-pressure spray gun. The samples were then heated at 100 °C in a forced-air drying oven for 3 h to accomplish complete curing, producing the test specimens.

### 2.5. Characterization

The emulsion solids content was measured as follows. The appropriate amount of emulsion was transferred in a watch glass and dried in a vacuum oven at 60 °C until the weight was constant. The solids content of the emulsion was determined as a percent of the dried weight compared with the initial weight.

(1) $$NV\%=\frac{W_{2}-W}{W_{1}-W}\times 100\%.$$

where NV-emulsion solid content, units: %; W-blank surface dish mass, units: g; W_1_-total mass of initial emulsion and surface dish, units: g; W_2_-total mass of final emulsion and surface dish, units: g.

The basic performance of WFPU film was evaluated in accordance with the following national standards: HG/T 3344-2012, GB/T 6739-2022, GB/T 9286-2021, and GB/T 1731-2020 [39,40,41,42]. The tests encompassed the water absorption rate, hardness, adhesion, and flexibility of the different WFPU film samples.

An FTIR spectrum of the samples was recorded using an FTIR spectrum analyzer (Vector-22, Bruker, Ettlingen, Germany). The wavelength was collected in the wavenumber range of 500 to 4000 cm^−1^ with a scan rate of 32 and a precision of 2 cm^−1^. The raw materials (including HFBMA, HPA, purified HFBMA-HPA, and WFPU) were coated directly onto the KBr discs. HFBMA and HPA did not require any treatment, and purified HFBMA-HPA and WFPU needed to be dried in a vacuum oven at 80 °C for 3 h before FTIR testing.

The WFPU emulsion was diluted with deionized water to approximately 0.02 wt% concentration, and the average particle size was determined with a nanoparticle analyzer (Zetasizer Pro, Malvern Co., Ltd., Malvern, UK). The final reported value was the average of three repeated measurements. The thinned particles of emulsion were stained using 2 wt% phosphotungstic acid solution and then observed under a transmission electron microscope (TEM, JEM-200CX, JEOL, Tokyo, Japan) to obtain TEM images.

Thermal characteristics of WFPU-n films were analyzed using a thermogravimetric analyzer (STA449-F3, Naichi Instrument Manufacturing Co., Ltd., Selb, Germany). The samples were treated by heating from 20 °C to 600 °C under Ar at a heating rate of 20 °C per min.

The surface morphologies of the samples were examined using a scanning electron microscope (QUANTA FEG 250, FEI Co., Ltd., Hillsboro, OR, USA) at a magnification of 2000× and an accelerating voltage of 10.0 kV. Before the SEM observations, all the samples were fixed on the sample holder using a conductive adhesive and then sputter-coated with a layer of thin gold under vacuum.

The WCA of the film surfaces was determined by a contact angle goniometer (JCY-2, Shanghai Fangrui Instrument Co., Ltd., Shanghai, China) employing drop shape analysis. Deionized water droplets with a volume of 5 μL were used for the measurements, which were conducted at ambient temperature. A micro-syringe was employed to deposit the deionized water drops. The reported contact angle values represented the average of five measurements. The surface energy of the WFPU films was calculated using Equations (S1), (S2), and (S3) [43].

The electrochemical workstation (model CHI600E, Shanghai Chenhua Instrument Co., Ltd., Shanghai, China) employing a conventional three-electrode setup was utilized to determine the electrochemical corrosion experiments. The samples were submerged in a 3.5 wt% sodium chloride solution, with a saturated calomel electrode, a platinum electrode, and the test specimen functioning as the reference, counter, and working electrodes, respectively. The potential was swept at a rate of 1 mV/s within the range of −800 mV to 800 mV relative to the open-circuit potential. The corrosion protection properties of the coatings were evaluated.

From the acquired Tafel polarization curves, the corrosion potential (E_corr_) and corrosion current density (I_corr_) of the samples were determined, which were then utilized to determine the anticorrosion performance of the different WFPU films. The protection efficiency (η) was calculated using Equation (S4). The polarization resistance (R_p_) was calculated from the Tafel plot using the Stern–Gray equation, as displayed in Equation (S5).

## 3. Results and Discussion

### 3.1. Basic Properties of the WFPU-n Films

Poor water resistance represents a significant challenge for WPU coatings. This is attributed to the incorporation of hydrophilic groups during the synthesis of the WPU emulsion. Furthermore, the low crosslinking density of the linear polyurethane structure leads to inadequate shielding against water molecules [44]. The water resistance of the film can be significantly enhanced by increasing the crosslinking density of the polyurethane molecular chains and introducing fluorine-containing groups. The water resistance of the films was characterized by the change in the water absorption rate of the films and the change in the appearance of the films after 24 h of immersion in deionized water, e.g., change in the color of the film and whether the film was cracked or not. As shown in Table 2, the maximum water absorption rate of WFPU-0 was 7.847% after submerging in deionized water for 24 h, while the water absorption rate of the other samples was less than that for WFPU-0, with WFPU-14 having the smallest water absorption rate of 2.962%. In addition, the color and appearance of all the samples were not changed, except for the WFPU-0 sample, which changed its color from transparent to light white after 24 h of submergence in deionized water.

Water absorption is one of the indicators of the water resistance of a material. Generally speaking, the greater the water absorption, the worse the water resistance of the material. The water absorption of the films exhibited a decreasing trend with the increasing addition of HFBMA-HPA. However, when the HFBMA-HPA addition exceeded 10.5 wt%, the change in water absorption became not significant. This was probably because of the limited ability of the fluorine-containing groups to enrich the surface. Once the fluorine level on the WFPU film surface reached a critical level, further increasing the HFBMA-HPA addition had little effect on the surface fluorine content. This suggests that the incorporation of fluorine-containing groups into the polyurethane molecular chains can effectively improve the water resistance of the films.

From Table 2, we can observe that the hardness of the WFPU samples was improved and adhesion and flexibility were at the highest level. This is because the epoxy groups introduced into WFPU can react with the isocyanate groups in the polyurethane to form a crosslinked structure [45]. This crosslinked structure can improve the hardness and adhesion of WFPU. Furthermore, the presence of hydroxyl groups in epoxy resin played a vital role in enhancing its adhesion. As a highly hydrophilic functional group, hydroxyl groups can form hydrogen bonds and other interaction forces with the hydrophilic functional groups on the surface of the substrate, thereby effectively improving the adhesion of WFPU coatings [46]. On the other hand, the PEG molecule is a linear polymer. The addition of PEG during the synthesis of polyurethane can improve the flexibility of the WFPU coating [47]. In summary, the addition of E-44 and PEG to WFPU can effectively improve the hardness, adhesion, and flexibility of the WPU coating.

### 3.2. FTIR Spectra of HPA, HFBMA, HFBMA-HPA, and WFPU-n

**Figure 3:**
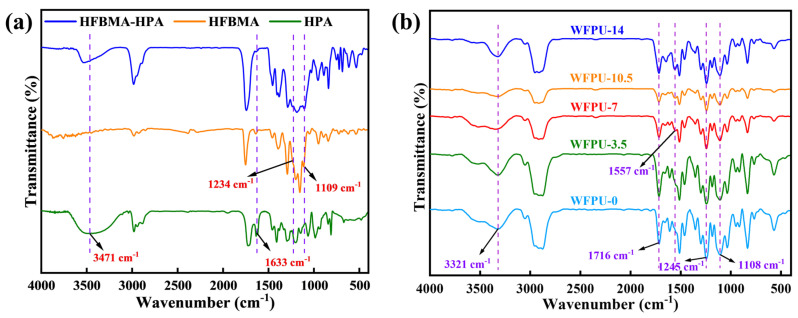
FTIR spectra of HPA, HFBMA, and HPA-HFBMA (**a**) and WFPU-n (**b**).

The FTIR spectra of HPA, HFBMA, and purified HFBMA-HPA provided the structural information, as depicted in Figure 3a. The absorption peak crests noticed at 3471, 2982, 2888, 1720, 1632, 1234, and 1109 cm^−1^ in the FTIR spectra corresponded to the O-H, CH_3_, and CH_2_ stretching vibration and C=O, C=C, and C-F stretching vibration, respectively [1,48].

The absorption peak at 3471 cm^−1^ in the FTIR spectrum of the HFBMA-HPA copolymer indicated the presence of -OH stretching vibration, which suggests that hydroxyl functional groups were introduced during the synthesis. The peaks observed at 1234 and 1109 cm^−1^ corresponded to the C-F stretching vibration [6,16], confirming the successful incorporation of the fluorine-containing monomer HFBMA in the copolymer. The disappearance of the C=C stretching vibration peak at 1633 cm^−1^ in the FTIR spectrum of the HFBMA-HPA copolymer indicates that the C=C double bonds in the HFBMA and HPA monomers were consumed during the free-radical copolymerization process, which also verifies the successful synthesis of the fluorine-containing segmented copolymer.

As depicted in Figure 3b, the characteristic absorption peak of -NCO group at 2270 cm^−1^ disappeared from the FTIR spectrum of WFPU emulsion [49], and the characteristic peaks of N-H bending vibration, N-H stretching vibration, and C=O telescoping vibration were observed at 3321, 1557, and 1716 cm^−1^, respectively [50,51], which indicates that the isocyanate group reacted with the hydroxyl group to form the urethane bond (-NHCOO-) and the polymerization reaction was complete. We also observed characteristic absorption peaks at 1615, 830, and 767 cm^−1^ for phenyl [22]. Based on the above FTIR analysis, we proved that we successfully synthesized polyurethane and introduced epoxy resin into the polyurethane molecular chain.

From the FTIR spectra of WFPU, it can be observed that the relatively strong absorption peaks at 1108 and 1245 cm^−1^ were attributed to the stretching vibrations of the CF_2_ and CF_3_ groups, which overlapped with the strong absorption peaks of the C-O-C groups [17,51,52], and it was also observed that the peaks here varied in magnitude and intensity for different WFPU samples. On the other hand, the peak at 1557 cm^−1^ became stronger and stronger with the increase in HFBMA-HPA addition. This was due to the increase in hydroxyl groups in the system, which reacted with the isocyanate group to form more urethane bonds, which also proved that the homemade fluorine-containing block copolymers were successfully introduced into the polyurethane backbone. In conclusion, the WFPU emulsion was successfully prepared.

### 3.3. Potentiodynamic Polarization Curves

**Figure 4:**
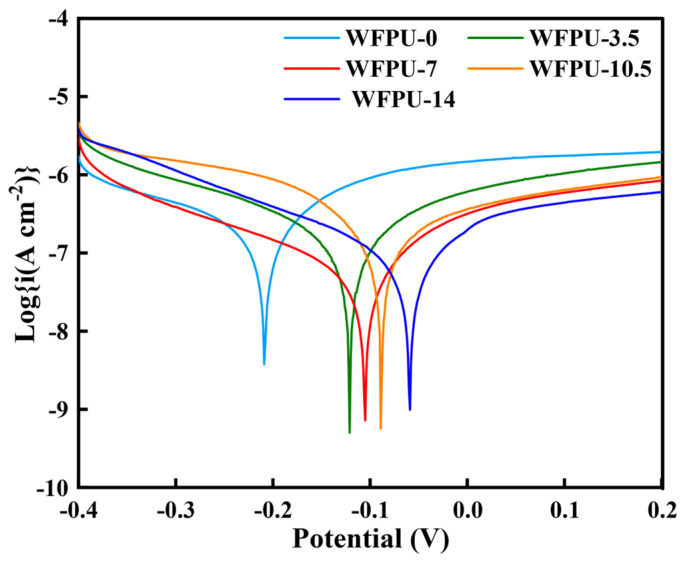
Tafel polarization curves of WPU-n films.

WPU can be used as a corrosion-resistant protective coating for metals. Interestingly, previous investigations reported that the incorporation of fluorine-containing groups into the WPU molecular structure can reduce metal corrosion [53,54]. Therefore, the introduction of fluorinated groups in WPU can improve the corrosion resistance of the coating and more effectively protect the substrate from corrosion. Subsequently, we measured the thickness of the varnish film using a micrometer (with an accuracy of 0.001 mm), which is shown in Table S2, and then selected samples of WFPU with varnish film thicknesses in the same range and with no defects in the varnish film. The effect of HFBMAHPA additions on the anticorrosive properties of WFPU-n coatings was investigated by potentiodynamic polarization studies using tinplate substrates coated with WFPU in 3.5% NaCl solution [55]. The Tafel polarization curves of the WFPU-n coatings are shown in Figure 4 and the corresponding E_corr_ and I_corr_ data are depicted in Table 3.

The values of E_corr_ and I_corr_ can be ascertained from the point of tangency between the cathodic and anodic polarization curves, with the x-axis representing I_corr_ and the y-axis representing E_corr_. It has been firmly established that I_corr_ is an indicator of corrosion rate of metal. A lower I_corr_ value signifies a less aggressive rate of corrosion. Meanwhile, E_corr_ is an indicator of the propensity for corrosion to occur; a more noble positive E_corr_ value suggests that the material is less susceptible to corrosion [56]. As depicted in Figure 4, the corrosion resistance of WFPU was significantly improved with the increase in HFBMA-HPA content, as evidenced by its higher E_corr_ and lower I_corr_ values compared with the WFPU-0 sample. Notably, the I_corr_ of the WFPU-14 sample decreased by two orders of magnitude in comparison with the WFPU-0 sample and η increased from 59.37% (WFPU-3.5) to 90.76% (WFPU-14) with the increase in fluorine content, as shown in Table 3.

The results revealed that the introduction of fluorine-containing groups into WPU can significantly improve the corrosion resistance of the coating and effectively protect the substrate from corrosion. This is due to the excellent water resistance of WFPU coatings, which effectively slows down the penetration of electrolyte into the interior of the coating and prolongs the time for the electrolyte to penetrate into the interior of the coating [57]. In addition, WFPU had a crosslinked structure. During the film formation process, a large number of fluorine-containing groups were confined inside the coating by the large molecular chains in WFPU. This coating structure made it difficult for corrosive media to penetrate the coating to reach the surface of the substrate, thus achieving the purpose of protecting the substrate from corrosion.

### 3.4. Thermogravimetric Analysis

**Figure 5:**
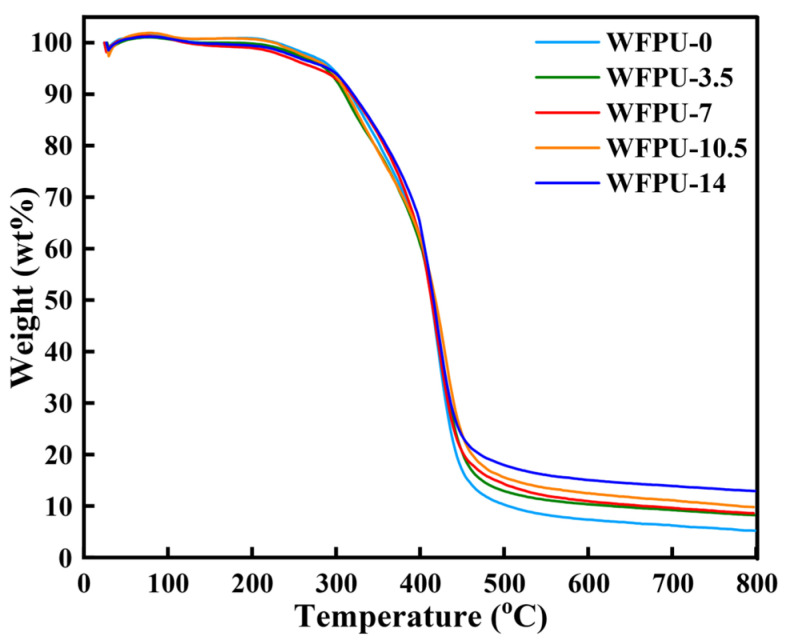
TGA curves of WPU-n films.

The thermal behavior of the WFPU-n sample can be elucidated by analyzing the TGA curve. As shown in Figure 5, the TGA curve showed a two-stage curve. This two-stage behavior can be explained by the composition of the WPU backbone, which consisted of isochrone diisocyanate (hard segment) and polyol (soft segment) [19]. The characteristic thermal decomposition temperatures of T_5%_, T_10%_, T_50%_, and T_80%_ for WFPU-n are shown in Table 4. The initial decomposition temperatures (T_5%_) of WFPU-0 and WFPU-14 were 295 °C and 289 °C, which was an increase of 136.6 °C and 130.6 °C, respectively, compared with the T_5%_ of one pure WPU (T_5%_ was 158.4 °C) sold on the market [58]. The T_50%_ of WFPU-0 and WFPU-14 were 413 °C and 417 °C.

The results showed that the initial decomposition temperature of WFPU samples decreased with increasing HFBMA-HPA content, which was due to the fact that small molecules that had not yet been incorporated into the crosslinked network and water within the membrane were first evaporated during the initial heating phase (0–300 °C) [16]. Then, in the first heat-loss phase (300–420 °C), we observed the thermal decomposition of the hard segments of WFPU. It was notable that the decomposition temperature of the WFPU-14 sample was always higher than that of the WFPU-0 sample in this stage, which was due to the small number of fluorine-containing groups that were confined by the macromolecular chains in the vicinity of the membrane’s hard segments and acted as shielding for the hard segments, thus increasing the thermal stability of the membrane. In the second heat-loss phase (420–520 °C), we observed the thermal decomposition of the soft segment of WFPU. It was noteworthy that the thermal decomposition temperatures of all the samples in this stage were higher than that of WFPU-0. This was because the intermolecular interactions such as van der Waals forces and hydrogen bonds were weaker in the soft segment, with weak intermolecular interactions reducing the restriction on the movement of fluorine-containing groups. The fluorine-containing groups could migrate to the membrane surface and enrich on the membrane surface to form a kind of thermal barrier, and the C-F (485 KJ/mol) bonds with high bonding energy played a stronger chemical shielding effect on the soft segments of WFPU [54]. In summary, the effect of HFBMA-HPA on the degradation process of the hard segments of WFPU chains was not too significant, but the C-F in the HFBMA-HPA copolymer with high bonding energy hindered the decomposition of the film during the second thermal decomposition stage. As the content of HFBMA-HPA increased, the hindering effect became more significant and the thermal stability of WFPU film was higher.

### 3.5. SEM Image of WFPU-n Films

**Figure 6:**
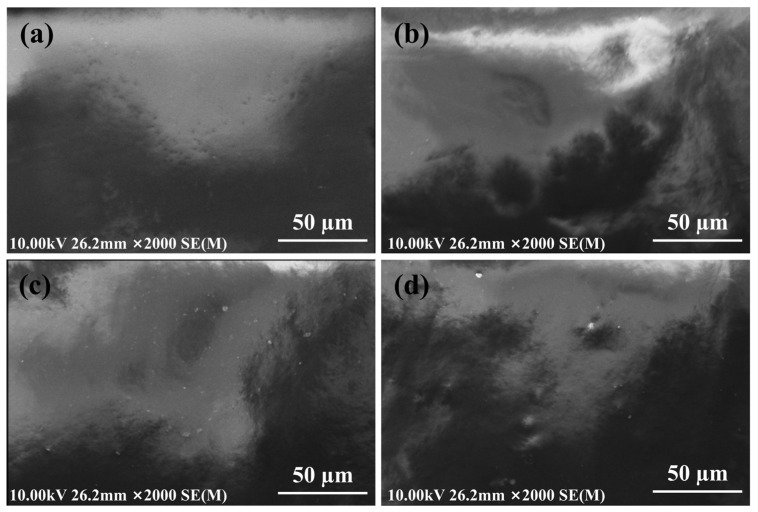
SEM images of WFPU-0 (**a**), WFPU-3.5 (**b**), WFPU-10.5 (**c**), and WFPU-14 (**d**).

Through SEM analysis, the surface morphology of the WFPU-0 (a), WFPU-3.5 (b), WFPU-10.5 (c), and WFPU-14 (d) film samples was examined, as depicted in Figure 6. Among the four film samples, the WFPU-0 film, which lacked the addition of HFBMA-HPA, exhibited a smoother surface morphology. This can be attributed to its simpler molecular chain structure and the near-complete evaporation of water after film formation, leading to reduced hindrance between the molecular chains.

As the addition of HFBMA-HPA increased, the surfaces of the WFPU-3.5, WFPU-10.5, and WFPU-14 film samples exhibited protrusions and small dots, with the WFPU-10.5 and WFPU-14 films displaying higher surface roughness. This can be explained by the introduction of HFBMA-HPA, which increased the crosslinking degree of the WFPU molecular chains. This increase in crosslinking impeded the mobility of the chains of molecules and enhanced hydrogen bonding interactions, resulting in the observed surface features. Furthermore, the WFPU samples contained fluorine-containing groups, whose surface energy was inherently low. In the process of film formation, fluorine-containing moieties often migrate and concentrate on the surface. This results in a considerable difference in surface free energy, which in turn accelerates the evaporation of water during the curing stage and subsequently increases the surface roughness of the film [59].

### 3.6. Particle Size of WFPU-n Emulsion

**Figure 7:**
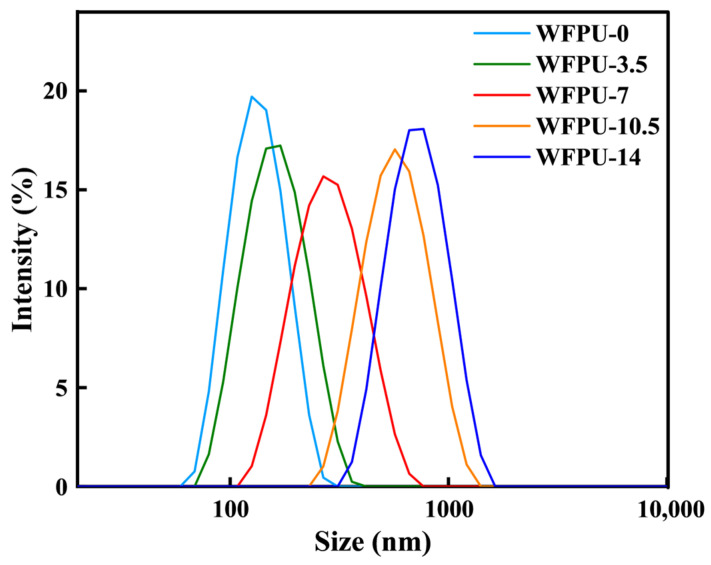
Particle size distribution of WFPU-n emulsions.

From Figure 7, we can observe that the WFPU emulsion particle size increased as the HFBMA-HPA content increased from 0% to 14%. It is well known that the content of hydrophilic chain segments (PEG) in WPU plays a major role in the size of the emulsion particles, and the less hydrophilic chain segments, the larger the emulsion particle size [19]. During self-emulsification, the hydrophobic chain segments in the latex particles are encapsulated by the hydrophilic chain segments. As the content of HFBMA-HPA increases, the hydrophobic chain segments increase, which leads to an increase in the particle size of the WFPU emulsion [2,16]. In addition, the fluorine-containing groups in the hydrophobic chain segments tend to enrich towards the surface, which makes the surface energy of WFPU emulsion particles decrease. This is also a good explanation of why the water absorption of the WFPU film decreased with the increase in fluorine content. In summary, we can conclude that the average particle size of WFPU emulsion increases with the increase in HFBMA-HPA content.

### 3.7. TEM Image of WFPU-n

**Figure 8:**
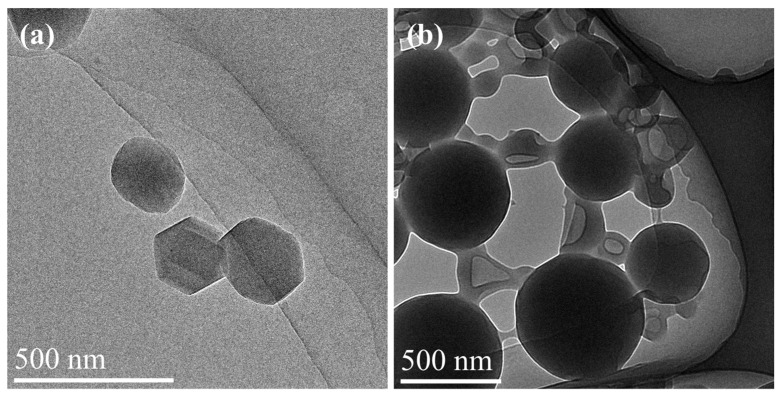
TEM images of WFPU-0 (**a**) and WFPU-10.5 (**b**).

Figure 8 presents the TEM observations of the latex particles for the WFPU-0 (a) and WFPU-10.5 (b) samples. The latex particles in the WFPU-0 sample, which lacked the addition of HFBMA-HPA, exhibited a regular spherical and uniform morphology, with a particle size of roughly 150 nm. In contrast, when the HFBMA-HPA content was 10.5 wt%, the latex particle size increased to around 450 nm.

The increase in the concentration of fluorinated chain segments enhanced the hydrophobicity of the WFPU film. This, in turn, led to the entanglement of the large hydrophobic chain segments during the emulsification process. As a result, the distribution of the latex particles was hindered, causing a certain degree of particle aggregation, which has been reported in the literature [60,61].

Conversely, with the increase in HFBMA-HPA content, the proportion of hard segments within the polymer also increased. This enhanced the interaction of hydrogen bonds, which in turn restricted the mobility of the WFPU chains. Consequently, this led to the aggregation of the latex particles [16]. Based on the particle size distribution graph, it can be observed that the particle size increased as the HFBMA-HPA content was raised. This observation was also consistent with the trend shown in Figure 8 and Table S1. In summary, as the HFBMA-HPA content in the WFPU latex was increased, a corresponding increase in the average particle size of WFPU latex particles was observed.

### 3.8. Surface Morphology of WFPU Films

The enriched elemental fluorine on the WFPU membrane surface has an impact on surface morphology [62]. For instance, it can contribute to a decrease in surface energy and a higher roughness of the film. This is an important factor affecting the surface properties of the film.

**Figure 9:**
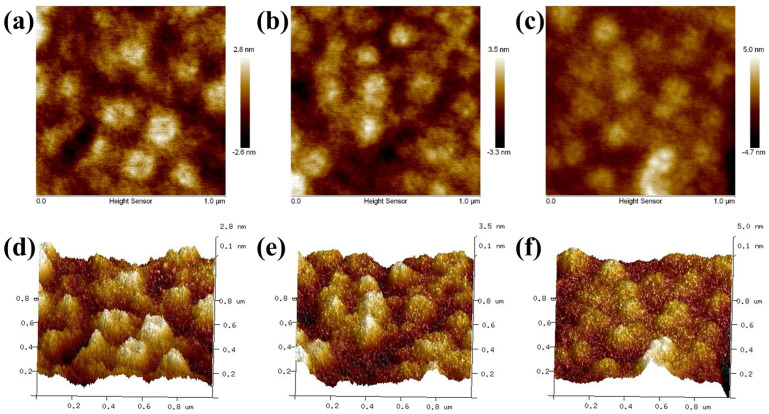
AFM phase images: WFPU-0 (**a**), WFPU-3.5 (**b**), and WFPU-10.5 (**c**). AFM topographies: WFPU-0 (**d**), WFPU-3.5 (**e**), and WFPU-10.5 (**f**).

The surface topography of the WFPU membrane was described and analyzed using AFM. The 2D and 3D images for the WFPU films are depicted in Figure 9 and their corresponding calculation results are depicted in Table 5. As the amount of added HFBMA-HPA was increased, the average surface roughness (R_a_) increased from 0.645 nm (WFPU-0) to 1.15 nm (WPFU-10.5), indicating that the surface roughness of the WFPU films became higher. Two- and three-dimensional AFM images revealed that the hard segments (the brighter regions) were aggregated on the membrane surface in the form of cylinders and spheres. This indicated the significant impact of incorporating HFBMA-HPA over the surface topography of the WFPU film. Furthermore, as the HFBMA-HPA content was increased, the interactions between the hard chain sections were enhanced, leading to greater interactions between the hard sections and more pronounced microphase segregation among the soft and hard chain sections.

In addition, during the film-forming process, as the water evaporated, the molecular chains containing hydrophobic groups gradually migrated to the solid–gas interface and aggregated on the thin film surface. This migration decreased the surface energy of the WFPU film and increased its surface roughness. Furthermore, some large-sized latex particles were restricted in their movement in the film formation process and did not aggregate on the surface of WFPU films, which could have contributed to the increase in surface roughness. In summary, the combination of the migration of fluorine-containing hydrophobic groups and the enhanced microphase separation between the soft and hard chain segments can be attributed as the main reason for the increased surface roughness of WFPU films.

**Figure 10:**
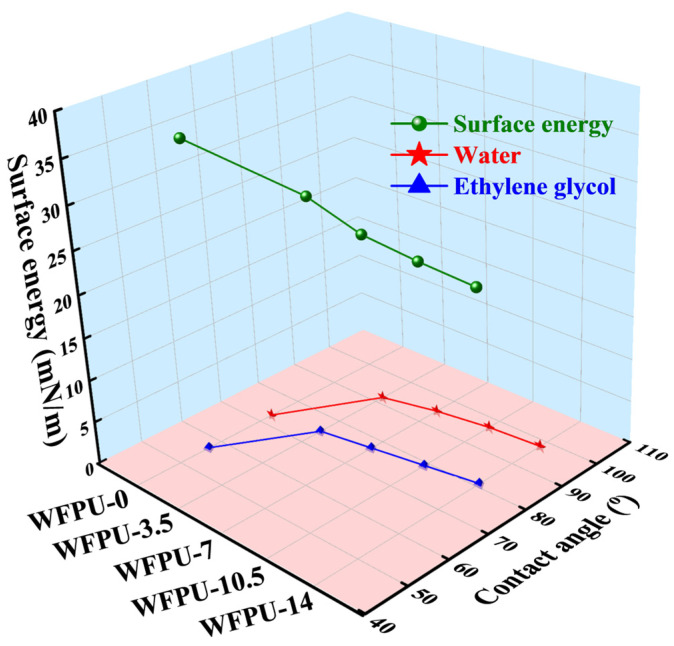
Relationships between water, diiodomethane and glycol contact angle, HFBMA-HPA content, and surface energy of WFPU films.

As depicted in Figure 10, the WCA of the WFPU film exhibited the same trend as the surface roughness, while the surface energy exhibited the opposite trend. This suggests that the enriched surface fluorine atoms on the membrane surface had a significant effect on both the WCA and surface energy of WFPU films.

## 4. Conclusions

In this study, HFBMA-HPA was successfully synthesized by free radical copolymerization, and HFBMA-HPA was used as a chain extender to prepare WFPU emulsions with excellent properties. The synthesis process was simple, environmentally friendly, and relatively low cost. This was because all the raw materials used in the synthesis process of the WFPU prepared in this study were commercially available. The toxic and hazardous substances generated during its life cycle from preparation to its natural degradation were less compared with solvent-based polyurethanes. There was no damage to the substrate during its use and the substrate could be coated several times. The water resistance, mechanical properties, thermal stability, and hydrophobicity of WFPU coatings increased with the content of HFBMA-HPA. When the content of HFBMA-HPA reached 14 wt%, the ƞ of WFPU-14 coating to the substrate was 90.76%. Thus, WFPU coating can contribute more in the field of corrosion protection. The WFPU coatings synthesized in this work showed significant improvement in hydrophobicity and mechanical properties, making them ideal candidates for large-scale or commercial outdoor protective coatings. However, despite the fact that researchers are continuously working on WFPU, its synthesis methods, structure, and properties are being extensively studied and show potential for applications in different fields such as coatings industry, textile industry, and biomedicine. The cost and environmental impact of WFPU synthesis are still challenges that need to be overcome. With the development of science and technology, researchers should explore the application potential of WFPU in emerging fields such as new energy and aerospace.

## Figures and Tables

**Table 1:** Synthesis formula of WFPU-n emulsion.

Sample	IPDI/g	PEG-200/g	HFBMA-HPA/g	PEG-4000/g	E-44/g
WFPU-0	5.16	2.20	0	10	22
WFPU-3.5	7.20	2.20	1.505	10	22
WFPU-7	9.64	2.20	3.300	10	22
WFPU-10.5	12.63	2.20	5.496	10	22
WFPU-14	16.32	2.20	8.220	10	22

**Table 2:** Basic properties of WFPU-n films.

Sample	Water Absorption (%)	Film Hardness	Adhesion	Flexibility (mm)
WFPU-0	7.847	3 H	0	2
WFPU-3.5	4.192	3 H	0	2
WFPU-7	3.614	3 H	0	2
WFPU-10.5	3.160	4 H	0	2
WFPU-14	3.062	4H	0	2

**Table 3:** Electrochemical parameters for WFPU-n coatings.

Sample	E_corr_ (mv)	I_corr_ (µA·cm^−2^)	b_a_ (mv)	b_c_ (mv)	R_p_ (MΩ cm^−2^)	Ƞ (%)
WFPU-0	−210	10.700	38.5	38.4	0.780	/
WFPU-3.5	−120	4.374	42.0	42.3	2.105	59.37
WFPU-7	−104	2.980	44.6	44.6	3.249	72.15
WFPU-10.5	−88	2.722	46.4	46.3	3.630	74.11
WFPU-14	−59	0.989	48.8	46.8	10.713	90.76

b_a_—anodic Tafel slope, b_c_—cathodic Tafel slope.

**Table 4:** TGA data of WFPU-n films.

Sample	T_5%_ (°C)	T_10%_ (°C)	T_50%_ (°C)	T_80%_ (°C)
WFPU-0	295	317	413	443
WFPU-3.5	287	309	416	451
WFPU-7	276	318	415	452
WFPU-10.5	290	313	418	462
WFPU-14	289	320	417	472

**Table 5:** Roughness of WFPU films.

Sample	R_a_ (nm)	R_q_ (nm)
WFPU-0	0.645	0.817
WFPU-3.5	0.802	0.994
WFPU-10.5	0.852	1.15

## Data Availability

The data are available upon request due to the volume of data and project limitations.

## Supplementary Materials

The following supporting information can be downloaded at: https://www.mdpi.com/article/10.3390/polym16182576/s1, Figure S1: WFPU synthesis and characterization overview diagram; The surface energy of the solid film can be determined using Equations (S1), (S2) and (S3); Table S1: particle size of WFPU samples; Table S2: Thickness of WFPU-n coatings.
