# Supplementary material for: Preparation and Characterization of Fluorinated Acrylate and Epoxy Co-Modified Waterborne Polyurethane
Source: Polymers (Basel). 2024 Sep 12;16(18):2576. doi: 10.3390/polym16182576 (PMC11435030; doi:10.3390/polym16182576)
Supplement: Supplementary file 1 [file polymers-16-02576-s001.zip › polymers-3155027-supplementary.pdf]

Supporting Information

Preparation and Characterization of Fluorinated Acrylate and Epoxy Co-Modified Waterborne Polyurethane

Yufei Zhao [1,2], Shuai Yang [3], Jianjun Zhang [1,2], Shaoxiong Xu [1,2], Jinhui Han [1,2] and Sude Ma [1,2,*]

[1] College of Materials Science & Engineering, Xihua University, Chengdu 610039, China
[2] Laboratory of Advanced Energetic Materials and Devices, Xihua University, Chengdu 610039, China
[3] Dongfang Electric Machinery Co., Ltd., Deyang 618000, China
* Correspondence: masude2007@163.com

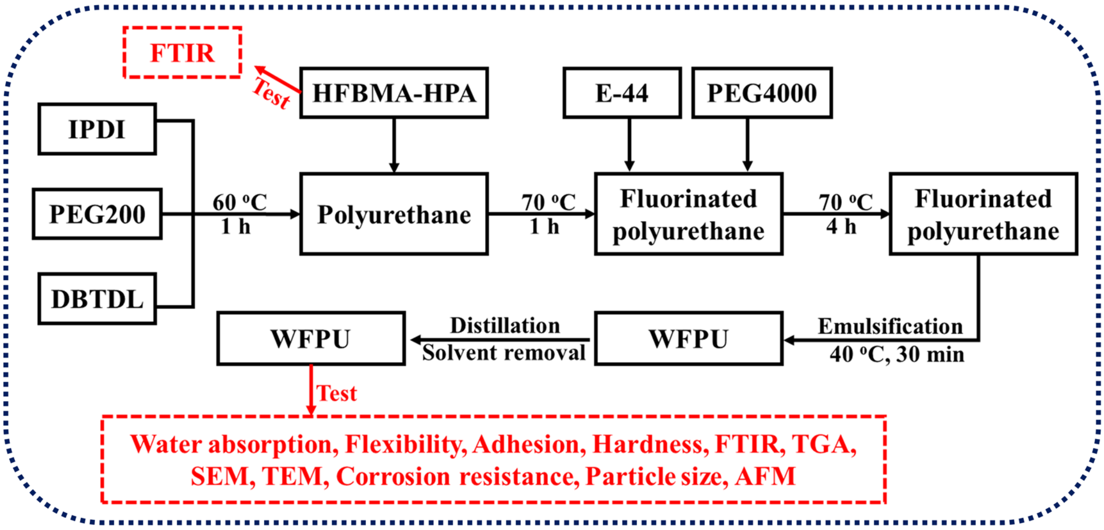

Figure S1: WFPU synthesis and characterization overview diagram

The surface energy of the solid film can be determined using equations S1, S2 and S3.

$$\gamma_s = \gamma_s^d + \gamma_s^p. \text{ S1}$$

$$\gamma_1(1+\cos\theta_1) = 4\left(\frac{\gamma_1^d\gamma_s^d}{\gamma_1^d+\gamma_s^d} + \frac{\gamma_1^p\gamma_s^p}{\gamma_1^p+\gamma_s^p}\right). \text{S2}$$

$$\gamma_2(1+\cos\theta_2) = 4\left(\frac{\gamma_2^d\gamma_s^d}{\gamma_2^d+\gamma_s^d} + \frac{\gamma_2^p\gamma_s^p}{\gamma_2^p+\gamma_s^p}\right). S3$$

where γ_s is the surface energy of solid film, γ_s^d is the dispersion force, and γ_s^p is the polarity force. θ_1, θ_2 are the CAs of water (1) and diiodomethane (2), γ_1^d, γ_1^p, γ_2^d and γ_2^p were 21.8, 51.0, 29.3, and 19.0 mN/m, respectively.

The protection efficiency (η) was calculated using equation S4.

$$\eta\ (\%) = 100\ \% \times \left(1 - \left(\frac{I_{coor,i}}{I_{coor,j}}\right)\right). \text{ S4}$$

where $I_{corr,\ i}$ and $I_{corr,\ j}$ values are corrosion current densities of the samples in the corrosive solution with and without the HFBMA-HPA, respectively.

The polarization resistance (Rp) is calculated from the Tafel plot using the Stern-Gray equation as displayed in equation S (5).

$$R_p = \frac{b_a b_c}{2.303(b_a+b_c)\times i_{coor}}. \text{ S5}$$

where I_{corr} is the corrosion current calculated by intersecting the linear sections of the anodic and cathodic curves, and b_a and b_c are the anodic and cathodic Tafel slopes (ΔE/ΔlogI), respectively.

Table S1: particle size of WFPU samples.

Sample	Particle Size (nm)
WFPU-0	129.4
WFPU-3.5	148.0
WFPU-7	252.4
WFPU-10.5	494.2
WFPU-14	573.0

Table S2: Thickness of WFPU-n coatings.

Sample	a (μm)	b (μm)	c (μm)	Average thickness (μm)
WFPU-0	22	20	21	21
WFPU-3.5	20	22	21	21
WFPU-7	20	21	22	21
WFPU-10.5	21	20	22	21
WFPU-14	22	20	21	21

Where a, b, and c are the thicknesses of the same WFPU sample measured at different locations, respectively.
